# Targeted immunotherapies for Graves’ thyroidal & orbital diseases

**DOI:** 10.3389/fimmu.2025.1571427

**Published:** 2025-03-12

**Authors:** Alan Chun Hong Lee, George J. Kahaly

**Affiliations:** ^1^ Division of Endocrinology and Metabolism, Department of Medicine, Queen Mary Hospital, The University of Hong Kong, Hong Kong, Hong Kong SAR, China; ^2^ Department of Medicine I, Johannes Gutenberg University (JGU) Medical Centre, Mainz, Germany

**Keywords:** targeted immunotherapies, autoimmune thyroid diseases, Graves’ disease, Graves’ hyperthyroidism, Graves’ orbitopathy

## Abstract

**Background:**

Graves’ hyperthyroidism and its associated Graves’ orbitopathy are common autoimmune disorders associated with significant adverse health impact. Current standard treatments have limitations regarding efficacy and safety, and most do not specifically target the pathogenic mechanisms. We aim to review the latest development of targeted immunotherapies in these two closely related disorders.

**Summary:**

Targeted immunotherapies of Graves’ hyperthyroidism have recently demonstrated clinical efficacy in early phase clinical studies. They include rituximab, an anti-CD20 monoclonal antibody which causes rapid B cell depletion; ATX-GD-59, an antigen specific immunotherapy which restores immune tolerance to thyrotropin receptor; iscalimab, an anti-CD40 monoclonal antibody which blocks the CD40-CD154 co-stimulatory pathway in B-T cell interaction; and K1-70, a thyrotropin receptor blocking monoclonal antibody. Furthermore, there have been major therapeutic advances in the management of Graves’ orbitopathy. Mycophenolate has a dual mechanism of action both inhibiting the proliferation of activated B & T cells as well as the mammalian target of rapamycin growth intracellular pathway. Rituximab appears to be effective in active disease of recent onset without impending dysthyroid optic neuropathy. Both tocilizumab (anti-interleukin 6 receptor monoclonal antibody) and sirolimus (mammalian target of rapamycin inhibitor) showed promise in glucocorticoid resistant active disease. Teprotumumab, an anti-insulin-like growth factor-1 receptor monoclonal antibody, demonstrated remarkable all-round efficacy across a wide disease spectrum. Linsitinib, a dual small molecule inhibitor of insulin-like growth factor-1 receptor and insulin receptor, displayed significant proptosis reduction in its phase 2b/3 study. Finally, Batoclimab, an anti-neonatal fragment crystallizable receptor monoclonal antibody, which blocks recycling of pathogenic thyrotropin receptor antibody, showed promising signals for significant proptosis reduction, disease inactivation, overall response, and improvement of quality of life.

**Conclusion:**

Therapeutic advances will continue to optimize our management of Graves’ hyperthyroidism and its associated orbitopathy in an effective and safe manner.

## Introduction

Graves’ disease (GD) is a common autoimmune thyroid disease affecting approximately 3% of women and 0.5% of men during their lifetime (1). GD is the most frequent cause of hyperthyroidism in iodine replete geographical areas. Recent population-based studies demonstrated an increased risk for all-cause mortality and acute cardiovascular diseases in patients with hyperthyroidism (2, 3). Graves’ orbitopathy (GO) is the most common extra-thyroidal manifestation of GD. The overall prevalence of GO in GD patients is 40% and GO appears to be slightly more prevalent among Asians when compared with Caucasians (45% vs 37%) (4). Disfigurement and disability resulting from GO carry a significant negative impact on patients’ quality of life and psychological well-being, as well as on our socioeconomic burden (5–7).

Current standard treatments for Graves’ hyperthyroidism (GH) and/or GO, including thionamide antithyroid drugs (ATD), radioactive iodine (RAI), thyroid surgery and systemic glucocorticoids, have been established since the mid-20^th^ century. It was not until the past two decades that novel therapeutic approaches have emerged which can better address the disease mechanisms underpinning GH and GO. This review will summarize the latest development of targeted immunotherapies in these two closely related autoimmune disorders.

## Graves’ hyperthyroidism: current management landscape

According to recent global surveys, 80-90% of clinicians chose ATD as the first-line treatment of new onset GH (8, 9), while RAI became much less popular than a decade ago (9). This observation is consistent with the latest recommendation by the European Thyroid Association (ETA) (1). Continuation or resumption of ATD was also preferred in around 60% of respondents in case of persistence or recurrence of GH (9). The major drawbacks of ATD include uncommon but severe toxicities (e.g. agranulocytosis, hepatotoxicity, ANCA-positive vasculitis, and acute pancreatitis [reported in methimazole users only (10)]), and high recurrence rate of 50% after a standard 12-18 month course of treatment (11, 12). In order to minimize risk of recurrence, long-term ATD for more than 60 months has been proposed as a safe and effective strategy which offers a high 4-year remission rate of 85% (13, 14). RAI is associated with significant risk of progression or *de novo* development of GO, especially in at-risk patients (15). Total thyroidectomy results in rapid cure of hyperthyroidism but surgical or anesthetic complications may occur. Lifelong thyroid hormone replacement is required following successful RAI ablation or total thyroidectomy. However, 5-10% of levothyroxine-treated hypothyroid patients reported dissatisfaction despite normal serum TSH level (16). Therefore, current standard treatments fail to achieve durable remission of hyperthyroidism without the need for long-term medications or causing iatrogenic hypothyroidism.

In recent years, several novel therapeutic strategies have been developed to treat GH by targeting the underlying immunopathogenic mechanisms: (1) B cell depletion; (2) restoration of immune tolerance to thyrotropin receptor (TSH-R); (3) interruption of B-T cell interaction via CD40-CD154 co-stimulatory pathway; and (4) interruption of TSH-R signaling.

## Targeted immunotherapies of Graves’ hyperthyroidism

### Rituximab

#### Background and scientific basis

Rituximab (RTX) is a chimeric murine/human anti-CD20 monoclonal antibody. By causing rapid depletion of B cells (from the stage of pre-B cells to mature and memory B cells, as well as short-lived plasma cells) in periphery and lymphoid organs (17), RTX is believed to inhibit B cell actions (e.g. antigen presentation, cytokine release) and reduce the synthesis of pathogenic autoantibodies through elimination of plasma cell precursors. Hence, RTX has been established as an effective treatment of B-cell malignancies (18) and various autoimmune disorders (19).

#### Summary of key clinical trials and clinical application

In a non-randomized pilot study (20), 20 GH patients were rendered euthyroid after about four months of methimazole treatment, and then assigned to RTX (375mg/m^2^ weekly for four doses) followed by ATD withdrawal or ATD withdrawal alone. Four of 10 patients in RTX group remained in remission with a median follow-up of around 2 years, while all patients in the observation group, who had similar baseline TSH-R antibody (TSH-R-Ab) levels, eventually relapsed by 14 months. Although both groups demonstrated similar decline in TSH-R-Ab levels measured by immunoassay, TSH-R stimulating antibody (TSAb) quantified by a bioassay reduced significantly only in RTX group (21). In a single-arm phase 2 study (22), 13 patients with relapsing GH received two doses of RTX one gram with a 2-week interval. Nine patients (69%) became euthyroid and remained in remission after a median follow-up of 18 months and they all achieved significant reduction in TSH-R-Ab levels. In another multicenter single-arm phase 2 study (23), 27 young patients (age 12-20) with new onset GH were given a single dose of RTX (500 mg) and a 12-month course of ATD. Thirteen patients (48%) remained in remission at 1 year after ATD withdrawal. Both remission and recurrence groups show similar baseline TSH-R-Ab levels. The authors concluded that adjuvant RTX may improve the likelihood of remission of GH in young patients whose remission rate was predicted at 20-30% after a 12-month course of ATD. Overall, the role of RTX in GH remains to be determined due to the absence of a formal randomized controlled trial (RCT). Its high cost and risk of severe side effects, including serum sickness-like reactions, iridocyclitis, polyarthritis, and inflammatory bowel disease (24), bring further concerns and limitations.

### ATX-GD-59

#### Background and scientific basis

Antigen-specific immunotherapy is an effective treatment for common allergic conditions, especially atopy and stinging insect hypersensitivity. Administration of the causative allergen (or antigen) at gradually increasing quantities results in desensitization and induction of immune tolerance (25). In recent decades, the growing understanding of the mechanisms of immune tolerance and autoimmunity has led to the development of antigen-specific approaches for the treatment of autoimmune diseases (e.g. type 1 diabetes (26, 27) and multiple sclerosis (28)). Approaches to antigen-specific therapy range from targeted deletion of autoreactive lymphocytes to tolerization of autoreactive T cells and active inhibition of autoimmune responses (29). For instance, immune tolerance can be restored through administering synthetic peptides (“apitopes”, i.e. antigen-processing independent T-cell epitopes) that mimic naturally processed CD4+ T cell epitopes (30). A mixture of two immunodominant apitopes based on the sequence of human TSH-R (ATX-GD-59) was sufficient to suppress both the T-cell and TSH-R-Ab response when administered in soluble form to HLA-DR3 transgenic mice immunized with human TSH-R (31).

#### Summary of key clinical trials and clinical application

In an open-label, single arm, phase 1 study (32), 10 patients with treatment-naïve mild to moderate GH (fT3 <= 15 pmol/L and fT4 <= 35 pmol/L) received all 10 doses of intradermal ATX-GD-59 over 18 weeks. Seven patients (70%) demonstrated complete (normalization of fT3 level, n=5) or partial response (reduction in fT3 and fT4 levels, n=2) at treatment end. Two patients remained euthyroid without ATD for a year after their last dose of ATX-GD-59. Reductions in serum fT4 and fT3 levels were significantly correlated with reductions in both TSH-R-Ab and TSAb levels. ATX-GD-59 was well tolerated and most adverse events (85%) were mild injection site reactions only. Antigen specific immunotherapy is an attractive therapeutic option in GH due to the absence of generalized immunosuppression and possibly durable drug-free remission. Future RCTs will explore its potential to achieve safe and effective long-term cure of GH.

### Iscalimab

#### Background and scientific basis

The breakdown of immune tolerance in GH generates autoreactive CD4+ helper T cells against TSH-R. T cell receptors interact with MHC Class II molecules of antigen presenting cells (primarily B cells) through which TSH-R peptides are presented. The interaction is followed by synthesis and presentation of CD154 (CD40 ligand, CD40L) on T cells, which binds to CD40 on B cells leading to co-stimulation of B cells. CD40-CD154 co-stimulatory pathway is essential for T cell dependent humoral immune response and plays an important role in the pathogenesis of GH by promoting autoreactive B cell activation, intrathyroidal germinal center function and TSH-R-Ab production. CD40 expression in thyroid tissue from GH patients was stronger than that in non-GH samples. CD40 agonist promoted thyroid tissue proliferation, thyroid hormone synthesis and thyroglobulin secretion *in-vitro* (33). Iscalimab is a Fc-silenced (nondepleting), fully human, pathway blocking anti-CD40 monoclonal antibody. It inhibited CD154-induced activation of human leukocytes *in-vitro*. Animal studies showed that it blocked primary and recall T cell-dependent antibody responses in nonhuman primates and abrogated germinal center formation without depleting peripheral B cells. Prolonged renal allograft survival in cynomolgus monkeys was also demonstrated (34). Iscalimab demonstrated favorable safety and efficacy profile in patients with Sjogren’s syndrome (35) and myasthenia gravis (36) according to recent phase 2 studies.

#### Summary of key clinical trials and clinical application

In a phase 2, single-arm proof-of-concept trial (37), 15 GH patients received 5 doses of intravenous (IV) iscalimab over 12 weeks, followed by a 24-week follow-up period. Almost complete CD40 engagement was evident for up to 20 weeks. Seven of 15 patients (47%, responders) achieved normal fT4/total T3 level by week 24. Overall, there was marked reduction of TSH-R-Ab levels (~40% at week 12 and ~70% at week 20), and 4 patients (27%) had normalization of TSH-R-Ab by week 20. All patients with baseline TSH-R-Ab less than 20 IU/L were responders. Four of 7 responders (57%) developed recurrence by week 36. Non-responders tended to have higher clinical score (based on pre-treatment age, goiter size, fT4 and TSH-R-Ab levels) which predicted recurrence of GH after a course of ATD (38). In addition, 2 responders who had GO at baseline showed ophthalmic improvement. Iscalimab was generally well tolerated without major safety signals. Further investigations of iscalimab in GH are warranted in view of its promising preliminary results.

### TSH-R blocking antibody

#### Background and scientific basis

Blocking the TSH-R from stimulation by TSAb represents the most direct strategy in managing GH. The human TSH-R blocking monoclonal antibody K1-70 was isolated in 2010 from a patient with high TSH-R-Ab level who initially presented with hyperthyroidism followed by hypothyroidism (39). In a subsequent *in-vivo* study, K1-70 resulted in a dose-dependent reduction of fT4 levels and inhibited the stimulatory effect of M22 on fT4 levels in rats (40). The same group reported the first experimental use of K1-70 which resulted in ophthalmic improvement (proptosis, inflammation), and possibly attenuation on tumor progression in a patient with co-existing follicular thyroid cancer and GH/GO (41).

#### Summary of key clinical trials and clinical application

The safety, tolerability, pharmacokinetics and pharmacodynamics of a single dose of K1-70 was evaluated in an open label, single arm, phase 1 study (42). Eighteen hyperthyroid or euthyroid ATD-treated GH patients received a single dose of intramuscular (IM, 0.2mg/1mg/5mg/25mg) or IV (50mg/150mg) K1-70. Significant effect on thyroid function was only observed in all 9 patients who received higher doses (25mg IM, 50mg/150mg IV) and they became hypothyroid on or before day 28 post-dose. Six of these 9 patients also showed various degree of ophthalmic improvement. Of note, remarkable proptosis reduction of 4-8mm by day 14-70 was evident in 3 patients who received K1-70 50/150mg IV. K1-70 was safe and well tolerated in all patients at all doses. K1-70 is believed to hold great promise in both GH and GO based on its sound mechanism of action, and future clinical trials are eagerly awaited.

### TSH-R small molecule ligands

#### Background and scientific basis

Multiple small molecule ligands have been developed to modulate TSH-R signaling and prevent TSH-R activation. *In-vitro* studies showed that TSH-R antagonists [NIDDK/CEB-52 (43); Org 274179-0 (44); ANTAG3 (45); VA-K-14 (46)] and inverse agonists [NCGC00161856 (47); NCGC00229600 (48)] were able to inhibit TSH/M22/TSAb mediated TSH-R activation, as measured by cAMP production and mRNA transcription for thyroglobulin, thyroperoxidase, sodium iodide symporter, and TSH-R. In mice treated with M22, ANTAG3 also successfully reduced serum fT4 level and mRNAs for sodium-iodide cotransporter as well as thyroperoxidase (45). Another TSH-R antagonist, SYD5115 (49), blocked M22/TSAb-mediated TSH-R activation in TSH-R-overexpressed Chinese hamster ovary cells and human orbital fibroblasts from GO patients. To date, TSH-R small molecule ligands have not been evaluated in human subjects.

**Figure 1:**
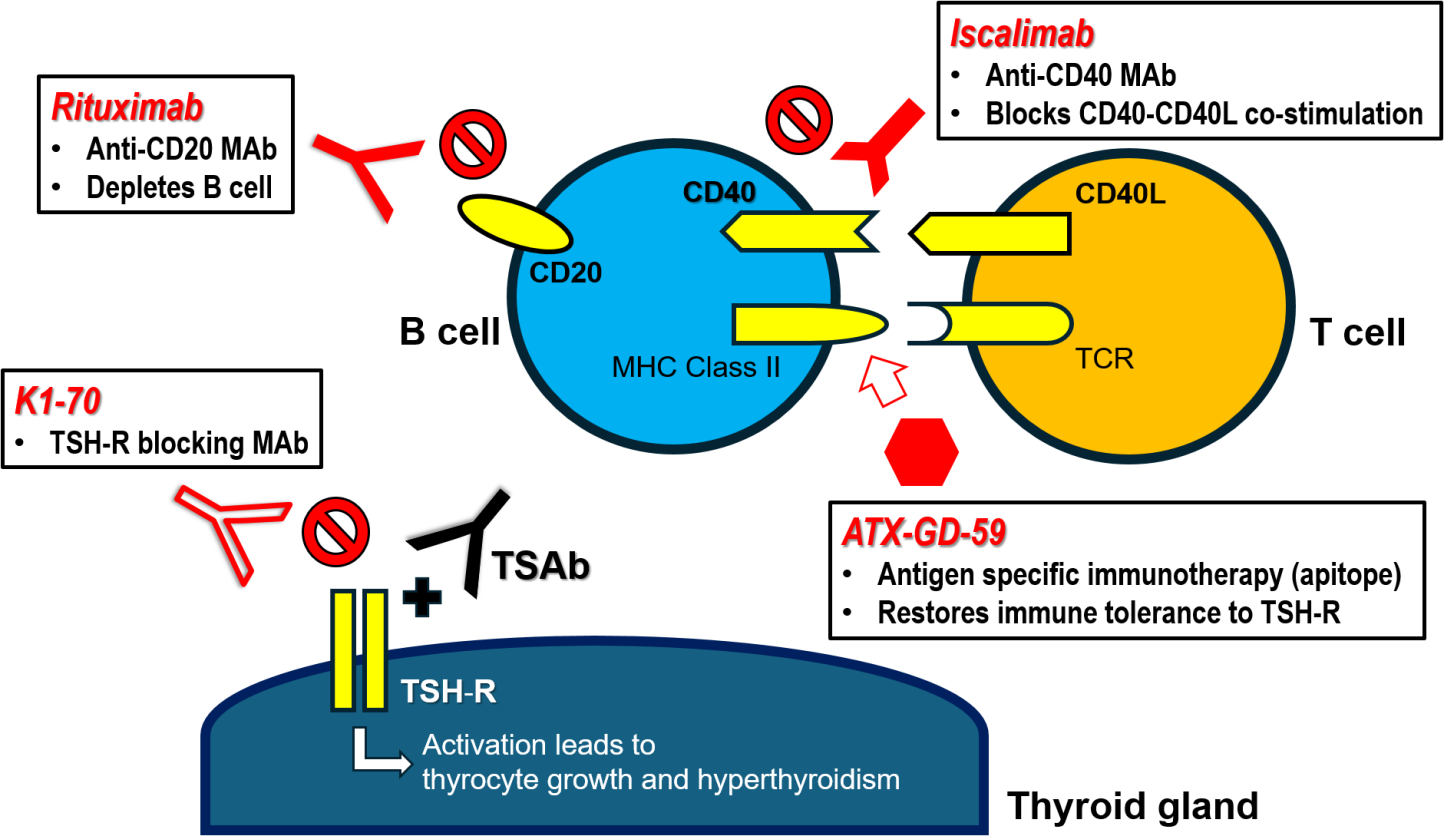
Targeted immunotherapies for Graves’ hyperthyroidism – mechanisms of action. CD40L, CD40 ligand (CD154); MAb, monoclonal antibody; MHC Class II, major histocompatibility class II molecule; TCR, T cell receptor; TSAb, thyrotropin receptor stimulating antibody; TSH-R, thyrotropin receptor.

The key clinical trials and mechanisms of action of targeted immunotherapies for GH are summarized in **Table 1** and **Figure 1**, respectively.

**Table 1:** Targeted immunotherapies for Graves’ hyperthyroidism: summary of key clinical trials.

Therapeutics	Mechanism of action	Study Design/Patient population	Key Findings	Common/Important toxicities
Rituximab	Anti-CD20 MAb	◼ Prospective, controlled, non-randomized study (20) ◼ 20 patients with untreated GH, rendered euthyroid after ~4 months of MMI, then withdrawn *➔ 10 RTX group (IV RTX 375mg/m^2^ weekly for 4 doses)* ➔ 10 Control group (Observation alone)	◼ 4/10 (40%) in RTX group remained in remission with median FU of 25 months ◼ 10/10 (100%) in control group relapsed by 14 months	◼ Serum sickness-like reactions ◼ Iridocyclitis ◼ Polyarthritis ◼ Inflammatory bowel disease
		◼ Prospective, single-arm phase 2 study (22) ◼ 13 patients with relapsing GH ◼ Intervention: 2 doses of IV RTX 1g with a 2-week interval	◼ 9/13 (69%) euthyroid and remained in remission after a median FU of 18 months (all responders achieved significant reduction in TSH-R-Ab)	
		◼ Prospective, multicenter, single-arm phase 2 study (23) ◼ 27 young patients (age 12-20) with new onset GH ◼ Intervention: single dose of RTX 500mg + ATD for 12 months	◼ 13/27 (48%) remained in remission at 1 year after ATD withdrawal (vs predicted remission rate of 20-30%)	
ATX-GD-59	Antigen-specific immunotherapy which restores immune tolerance to TSH-R	◼ Open-label, phase 1, single-arm study (32) ◼ 10 patients with untreated GH ◼ Intervention: 10 doses of intradermal ATX-GD-59 over 18 weeks	◼ 5/10 (50%) euthyroid by week 18; 3 of them remained relapse free for a year after last dose of study drug ◼ 2/10 (20%) had reduction in fT3/fT4 ◼ Response in serum thyroid hormones correlated with reductions in both TSH-R-Ab and TSAb levels	◼ Mild injection site reactions
Iscalimab	Anti-CD40 MAb blocking CD40-CD154 co-stimulatory pathway	◼ Open-label, phase 2, single-arm, proof-of-concept study (37) ◼ 15 patients with untreated GH ◼ Intervention: 5 doses of IV iscalimab (10mg/kg) over 12 weeks	◼ 7/15 (47%) euthyroid by week 24; 4/7 (57%) responders relapsed by week 36 ◼ Marked reduction in TSH-R-Ab levels; 4/15 (27%) achieved normal TSH-R-Ab levels by week 20	◼ Nil
K1-70	TSH-R blocking MAb	◼ Open label, single arm, phase 1 study (42) ◼ 18 ATD-treated GH patients ◼ Intervention: Single dose of K1-70 of 6 different regimens (IM 0.2/1/5/25mg; IV 50/150mg)	◼ All patients on higher dosages (9/18) had significant effect on thyroid function (25mg IM; 50/150mg IV) and all became hypothyroid within 1 month ◼ 6/9 (67%) of these patients showed GO improvement ◼ Significant proptosis reduction of 4-8mm in 3 patients on higher dosages (50mg/150mg IV)	◼ Nil

ATD, antithyroid drug; FU, follow-up; GH, Graves’ hyperthyroidism; GO, Graves’ orbitopathy; IM, intramuscular; IV, intravenous; MAb, monoclonal antibody; RTX, rituximab; TSAb, thyrotropin receptor stimulating antibody; TSH-R, thyrotropin receptor; TSH-R-Ab, thyrotropin receptor antibody.

## Graves’ orbitopathy: current management landscape

European Group on Graves’ Orbitopathy (EUGOGO) (15) and American Thyroid Association (ATA)/European Thyroid Association (ETA) (50) have published comprehensive management guidelines on GO. Key general measures include referral to combined thyroid eye clinics for multidisciplinary care, smoking cessation, restoration/maintenance of stable euthyroidism, judicious use of RAI (with steroid prophylaxis in high-risk patients), and local treatment for dry eye syndrome as well as corneal exposure. Selenium supplementation should be given to patients with mild active GO of recent onset, as it improves eye manifestations and quality of life, as well as prevents progression to more severe forms (15, 51). A recent small RCT suggested that selenium also improved eyelid aperture in selenium-replete patients with inactive moderate-to-severe GO (52). EUGOGO recommends the combination of intravenous glucocorticoid (IVGC) and mycophenolate as the first-line treatment of active moderate-severe GO (15, 53). High dose IVGC is the mainstay of treatment for dysthyroid optic neuropathy (DON) and urgent surgical orbital decompression should be considered if response is poor or absent within 1-2 weeks (15, 50). In inactive and stable disease, rehabilitative surgery may be required if there is residual functional impairment or cosmetic concerns (15, 50).

There are several limitations of IVGC therapy. The overall response rates were approximately 70% and 50% only in active moderate-severe GO and DON, respectively (12, 54, 55). Some responders develop disease reactivation after steroid withdrawal. The addition of mycophenolate or atorvastatin improved the overall response (53, 56). IVGC mainly improves inflammatory features, but is ineffective in reversing orbital tissue remodeling, e.g. proptosis, ocular dysmotility and diplopia (57). Severe cardiovascular and liver toxicities occurred infrequently (15, 57). Over the past decade, advances in our understanding of pathogenesis have fostered multiple clinical trials in GO which evaluated the role of immunotherapies targeting B/T-cells, pro-inflammatory cytokine interleukin-6 (IL-6), TSH-R and insulin-like growth factor-1 receptor (IGF-1R) signaling, as well as TSH-R-Ab.

## Targeted immunotherapies of Graves’ orbitopathy

### Mycophenolate

#### Background and scientific basis

Mycophenolic acid (MPA) is a competitive reversible inhibitor of inosine monophosphate dehydrogenase (IMPDH), which governs the rate-limiting step of *de novo* synthesis of guanosine nucleotides in activated lymphocytes. As lymphocytes cannot synthesize guanosine nucleotides via the salvage pathway, MPA exerts dual antiproliferative effect on both B and T cells (58). In addition, mycophenolate demonstrated antifibrotic property by inhibiting fibroblast proliferation and functions via both IMPDH-dependent and IMPDH-independent pathways (59–64). Mycophenolate has also been shown to inhibit the phosphatidylinositol 3-kinase (PI3K)-Akt-mammalian target of rapamycin (mTOR) pathway, an important downstream signaling pathway of orbital fibroblasts, in a rat model of epilepsy (65).

#### Summary of key clinical trials and clinical application

In a multicenter RCT (53), 164 patients with active moderate-to-severe GO were randomized to weekly IVGC 4.5g for 12 weeks or a combination of IVGC for 12 weeks and mycophenolate sodium 720mg daily for 24 weeks. While the primary endpoints (response rate by composite ophthalmic index at week 12, relapse rates at weeks 24/36) were not met, the combination group demonstrated superior response rate at week 24 (71% vs 53%) and sustained response rate at week 36 (67% vs 43%). *Post-hoc* analysis further showed that the combination group had more significant improvement in clinical activity score (CAS), swelling of eyelid or caruncle, orbital pain, chemosis, downgaze duction and elevation, as well as Graves’ orbitopathy quality of life questionnaire (GO-QoL) visual functioning subscale. The safety and tolerability of mycophenolate in GO were systemically evaluated (66–68). Most side effects in the combination group were mild and there was no treatment related serious adverse event. Gastrointestinal intolerance was slightly more frequent among patients on combination therapy. The addition of low dose mycophenolate did not significantly increase the risks of serious infection, hepatotoxicity and cytopenia. Therefore, EUGOGO recommends the combination of IVGC and low dose mycophenolate, which is safe and affordable, as the first-line treatment of active moderate-to-severe GO (15).

### Rituximab

#### Background and scientific basis

Apart from peripheral B cell depletion, complete (or near complete) intra-orbital depletion of both B and T cells after RTX therapy has been confirmed in multiple reports (69). Since the clinical improvement of GO post-RTX does not necessarily parallel significant decline in TSH-R-Ab level, the efficacy of RTX may be attributed to elimination of other B cell functions beside autoantibody production (e.g. cytokine production, antigen presentation, B-T cell co-stimulation, etc.) (17).

#### Summary of key clinical trials and clinical application

Two RCTs evaluated the efficacy of RTX in patients with active moderate-to-severe GO, but they demonstrated essentially opposite outcomes. The Italian trial (70) compared RTX (1g twice at 2-week interval or single dose of 500mg) with weekly IVGC (12 doses, cumulative dose 7.5g). All patients in RTX group achieved disease inactivation at week 24 (68.7% in IVGC group) and none developed reactivation (31.2% in IVGC group). There was no significant difference in Gorman score for diplopia and proptosis between the two groups, but better ocular motility (in terms of total degree of ductions) and less requirement of rehabilitative surgery were observed in RTX group up to 18 months post-treatment. However, RTX was not superior to placebo regarding CAS and other secondary endpoints in the US trial (71).

In a subsequent joint *post-hoc* analysis, several key differences in baseline characteristics, namely younger age (mean 51.9 vs 57.6), lower TSH-R-Ab (mean 10.7 vs 28.1 IU/L) and shorter duration of GO (mean 4.5 vs 30 months), may explain the favorable treatment outcomes in the Italian trial (72). The absence of B cell orbital infiltration in some GO patients may explain why they failed to respond to RTX (69, 73). More recent retrospective series also found reduced efficacy of RTX in glucocorticoid resistant active moderate-to-severe GO with long disease duration (74, 75). Two single-arm studies suggested that very low dose RTX (100mg once) with or without a short course of IVGC was also effective and better tolerated than standard RTX/IVGC regimens in patients with short duration of GO (76, 77) but the absence of control groups precludes head-to-head comparison. A few RTX-treated patients in RCTs developed transient deterioration or even frank DON, which could be explained by cytokine release syndrome because of massive lysis of intra-orbital B cells, leading to further oedema and expansion of orbital tissue (72). Hence, RTX is only considered as a second-line treatment of active moderate-to-severe GO of relatively short duration (e.g. less than 9 months) without potential risk of DON according to latest guidelines, and its role is mainly on disease inactivation and prevention of relapses, but not on diplopia or proptosis improvement (15, 50).

### Tocilizumab

#### Background and scientific basis

IL-6 is a potent pro-inflammatory cytokine implicated in the pathogenesis of various autoimmune diseases such as rheumatoid arthritis, multiple sclerosis and systemic lupus erythematosus. TSH-R activation (by TSH or M22) increased expression and production of multiple chemo attractants including IL-6 from GO orbital fibroblasts (78–80). In turn, IL-6 enhanced expression of TSH-R in GO orbital fibroblasts (81). IL-6 supported B cell differentiation and synthesis of autoantibodies (82). IL-6 suppressed regulatory T cell induction while promoting the development and functions of Th17 cells, a recently identified CD4+ T cell subset which plays an important pathogenic role in GO (83–85). Higher serum levels of IL-6 were noted in GD patients with GO than those without GO (86). Greater lacrimal levels of IL-6 were also shown in GO patients compared to healthy controls and they positively correlated with CAS (87, 88). Therefore, inhibiting the IL-6 pathway represents a reasonable strategy in GO.

#### Summary of key clinical trials and clinical application

Tocilizumab (TCZ) is a fully human IgG1 anti-IL-6 receptor monoclonal antibody. A small RCT evaluated the efficacy of TCZ in patients with glucocorticoid-resistant active moderate-to-severe GO (89). 32 patients were randomized to TCZ (8mg/kg intravenously once every 4 weeks for 4 doses) or placebo. 93.3% and 86.7% in TCZ group showed improvement in CAS by at least 2/10 at week 16 and week 40, respectively (vs 59% in placebo group at both weeks). CAS of less than 3/10 was achieved in 86.7% and 80% in TCZ group at week 16 and week 40, respectively (vs 35.2 and 47.1% in placebo group). Overall, the clinical benefits were improvements in soft tissue involvement and CAS, but it did not have significant effect on proptosis and diplopia. The potent anti-inflammatory effect of TCZ in active GO appears promising, although the small sample size and the arbitrary definitions of glucocorticoid resistance limited its interpretation and application. Thereafter there has been great enthusiasm about applying TCZ in glucocorticoid resistant GO. A recent systematic review and meta-analysis (90) analyzed 12 studies with 219 patients who received TCZ for glucocorticoid resistant active moderate-to-severe GO, and most were single-arm prospective or retrospective cohort studies. TCZ demonstrated significant improvement in CAS (effect size 0.98, mean reduction 4.6), proptosis (effect size 0.5, mean reduction 2.04mm), diplopia (effect size 0.48), and TSH-R-Ab levels (mean reduction 10.62 IU/L).

TCZ is overall safe and well tolerated. Common side effects of TCZ include mild neutropenia, hypercholesterolemia and transient rise in liver enzymes. Severe toxicities (e.g. diverticulitis and gastrointestinal perforation, serious infection) were rare. Based on the promising findings from multiple observational studies, TCZ can be employed for disease inactivation in glucocorticoid-resistant active moderate-to-severe GO (15, 50). Future RCTs will further define its role as 1^st^ line (NCT04876534; versus IVGC 4.5g) or 2^nd^ line/rescue treatment (NCT01297699; versus placebo) in active GO.

### Sirolimus

#### Background and scientific basis

Sirolimus is a mammalian target of rapamycin (mTOR) inhibitor widely used in the field of transplantation medicine. Given its antiproliferative action, it is also useful in several diseases characterized by abnormal cellular proliferation (e.g. tuberous sclerosis, autosomal dominant polycystic kidney disease, lymphangioleimyomatosis). As mTOR is an integral component of the phosphatidylinositol 3-kinase (PI3K)-Akt-mTOR pathway which mediates downstream signaling of IGF-1R (91), sirolimus may play a role in GO management. An *in-vitro* study showed that rapamycin/sirolimus significantly reduced fibrosis in orbital fibroblasts from GO patients and this effect was independent of, and in addition to its immunosuppressive effect (92). In a GO mouse model, rapamycin significantly decreased the incidence of GO. This was accompanied by the reduction of both CD4+ cytotoxic T-cells and the reduction of orbital inflammation, adipogenesis, and fibrosis. CD4+ cytotoxic T-cells from patients with active GO showed upregulation of the mTOR pathway, while rapamycin decreased their proportions and cytotoxic function (93).

#### Summary of key clinical trials and clinical application

The efficacy of sirolimus in GO was first described in 2 case reports of patients with refractory DON and ocular dysmotility (94, 95). In a prospective comparative case series, the combination of IVGC/orbital radiotherapy and sirolimus resulted in better improvement of diplopia when compared with combination of IVGC/orbital radiotherapy and mycophenolate (96). An observational study compared the efficacy of low dose sirolimus (2mg orally on first day, followed by 0.5mg daily for 12 weeks) and a second course of 4.5g IVGC as second-line treatments for 30 patients with glucocorticoid resistant active moderate-to-severe GO (97). When compared to IVGC, significantly more patients in sirolimus group achieved overall response (86.6% vs 26.6%), proptosis response (80% vs 13.3%) and CAS response (86.6% vs 33.3%) by week 24. There was also a trend towards better diplopia response in sirolimus group, but it did not reach statistical significance (63.6% vs 23%, p = 0.052). Patients treated with sirolimus reported significantly better GO-QoL scores (total and visual functioning subscale). No serious adverse events were observed. However, all treatment outcomes, except for CAS response, did not differ between the 2 groups at week 48 (98). The optimal dose and duration of sirolimus therapy remains to be determined in future RCTs.

### Teprotumumab

#### Background and scientific basis

TSH-R is the principal autoantigen in GH/GO and its stimulation leads to activation of GO orbital fibroblasts (GO-OF). Both TSH-R and IGF-1R were over-expressed in GO-OF. They formed a physical and functional complex, whose activity was important for TSH-R downstream signaling. TSH-R and IGF-1R were in proximity in a signalosome, and β-arrestin 1 acted as a scaffold to mediate receptor crosstalk. Simultaneous activation of both TSH-R and IGF-1R synergistically increased hyaluronic acid (HA) secretion by GO-OF. *In-vitro* studies showed that TSH-R antagonist (ANTAG3) fully suppressed M22 induced HA secretion by GO-OF regardless of M22 concentration. In contrast, linsitinib (IGF-1R kinase inhibitor) and 1H7 (IGF-1R blocking antibody) fully antagonized HA secretion induced by M22 at low concentration, but their efficacy diminished at high concentration of M22. The combination of ANTAG3 and linsitinib/1H7 synergistically suppressed HA secretion. The stimulation of TSH-R activates two signal transduction pathways, one being IGF-1R independent and the other IGF-1R dependent (i.e. TSH-R/IGF-1R crosstalk pathway), resulting in more intense activation of GO-OF (91, 99, 100). Teprotumumab (TPT) is a fully human IgG1 monoclonal blocking antibody. TPT was initially designed as an anti-cancer therapy but its development program was discontinued in 2009 for commercial reasons. TPT was subsequently repurposed for potential use in GO based on preclinical data. It has been proven that inhibition of TSH-R/IGF-1R crosstalk via β-arrestin 1 was the mechanism explaining the remarkable efficacy of TPT (101). Reversal of tissue modelling may also be attributed to GO-OF/adipocyte death via the cell-extrinsic pathway of apoptosis because of inhibition of IGF-1R signaling (102).

#### Summary of key clinical trials and clinical application

The safety and efficacy of TPT was evaluated sequentially in two randomized, placebo-controlled multicenter trials, which recruited a total of 171 patients with active moderate-to-severe GO (103, 104). Both trials had almost identical design and patients were randomly assigned to TPT (84 patients; once every 3 weeks intravenously for 8 doses over 24 weeks) or placebo (87 patients). The primary endpoint of the initial phase 2 study was a composite of ≥2 point CAS reduction and ≥ 2mm proptosis reduction at week 24, whereas the subsequent phase 3 study only focused on proptosis reduction as the primary outcome. The integrated outcomes from both trials were analyzed and summarized below (105).

TPT demonstrated remarkable all-round efficacy in terms of disease activity, severity, and QoL when compared to placebo. At week 24, 62% in TPT group achieved disease inactivation with CAS 0/1 (vs 22% in placebo group). The mean CAS reduction in TPT group was 3.99 (vs 2.31). Proptosis response was observed in 77% among TPT-treated patients (vs 15%) and the mean proptosis reduction was 3.14mm (vs 0.37mm). Proptosis response occurred early at week 6 in most patients. The proptosis response was similar across all subgroups stratified according to age, sex, smoking status, GO duration, baseline CAS, and baseline TSH-R-Ab levels. An overall response (≥2 point CAS reduction AND ≥ 2mm proptosis reduction) was observed in 74% of patients in TPT group (vs 14%). Diplopia response (≥1 grade improvement from baseline) was significantly more prevalent in TPT group (70% vs 31%), and 53% in TPT group even reported resolution of diplopia (vs 25%). Among patients with constant diplopia at baseline, 71% in TPT groups experience ≥1 grade improvement (vs 18%). TPT group had a greater improvement in GO-QoL (mean total/visual functioning/appearance subscales) compared to placebo group.

Patients in the previous phase 3 study (104) who received placebo, were proptosis non-responders or were initially proptosis responder but developed disease flare (≥ 2mm increase in proptosis, ≥ 2 point increase in CAS, or both) entered an open-label extension study where they were either treated with TPT for the first time (previous placebo patients, n = 37) or re-treated with the second course of TPT (non-responders, n = 5 or flare, n = 8) (106). Thirty-three of 37 patients (89.2%) became proptosis responders with a median time to response of 6.4 weeks and a mean reduction of 3.5mm. Diplopia response, CAS response and overall response were observed in 60.9%, 65.6%, and 78%, respectively. Two of 5 non-responders (40%) showed proptosis response after re-treatment. Five of 8 patients (62.5%) who flared responded again. The findings suggested that GO with longer disease duration responded to TPT in a similar fashion as those treated earlier (12.3 vs 6.4 months), and patients with initial suboptimal response or relapse may benefit from additional TPT therapy.

The treatment responses of TPT were durable from a recent extended follow-up outcome analysis of 112 patients from the above three trials (107). At 1 year after the last dose of TPT, 91.2% (vs 86.6% at week 24), 89.5% (vs 91.1%), 72.9% (vs 70.2%), 67.9% (vs 86.6%), and 66.1% (vs 76.8%) of patients were responders for CAS, composite outcome, diplopia, proptosis, and overall response, respectively. Over 2 years following TPT therapy, 18% of patients underwent additional GO therapy (e.g. systemic steroid, rehabilitative surgery). In another retrospective series of 119 TPT-treated patients, the re-treatment rate was 24% and older age was the only risk factor identified (108). Additionally, TPT has also demonstrated efficacy in GO of long duration and low disease activity. In a randomized, double-masked, placebo-controlled trial (109), 62 patients with significant proptosis, long GO duration of 2-10 years, low CAS ≤1 and stable disease for ≥1 year were randomized to TPT and placebo. A significantly greater proportion of patients in TPT group had a proptosis response (62% vs 25% in placebo group) and the mean proptosis reduction was 2.41mm in TPT group (vs 0.92mm in placebo group).

While over 80% of participants in the two pivotal RCTs were Caucasian (103, 104), a recent small RCT of similar study design evaluated TPT exclusively in Japanese patients with active moderate-to-severe GO and 54 patients were randomized to TPT and placebo (110). At week 24, TPT group demonstrated superior efficacy in terms of proptosis reduction (89% vs 11%; mean reduction 2.36mm vs 0.37mm), disease inactivation (59% vs 22%), overall response (89% vs 11%), diplopia resolution (e.g. 50% vs 20%) and GO-QoL. Hence, the efficacy of TPT is consistent across different ethnic groups.

TPT was generally well tolerated, and most adverse events were mild to moderate in severity (103–105). The most common side effect was muscle spasm (18%). Two important adverse events, namely hyperglycemia and hearing dysfunction, were noted in around 10% of TPT-treated patients in RCTs and deserve special attention. In an observational longitudinal study (111), 22 of 42 (52%) TPT-treated patients developed hyperglycemia, although 19 of 22 cases were graded as mild to moderate. Age, pre-existing diabetes, Hispanic and Asian race/ethnicity were significant risk factors for hyperglycemia. Patients with pre-existing prediabetes and diabetes had a significant mean increase in HbA1c at 3 months by 0.7% and 1.3%, respectively. Only eight of 22 patients who developed hyperglycemia returned to baseline glycemic status at 1 year post-treatment. The risk of TPT-related hyperglycemia appears to be higher in real-world practice. Therefore, patients who receive TPT should optimize their glycemic control before treatment, undergo close glycemic monitoring during treatment and receive timely management of hyperglycemia if it arises (112). Real-world data (113–116) suggested that around 30% of TPT users reported hearing loss or other otologic symptoms (e.g. tinnitus, autophony, ear fullness). The mean symptom onset was after around 3 to 4 TPT infusions and these otologic complaints were persistent in approximately 30-50% of cases. In a prospective study of 52 TPT-treated patients with serial audiometry testing (116), baseline hearing loss was documented in 20/52 (38%) patients. The risk of post-treatment hearing dysfunction was significantly higher in older patients and those with baseline hearing loss (9/20, 45%) compared with those having normal baseline hearing (1/32, 3%). 4/20 (20%) patients had persistent hearing dysfunction at 6 months post-treatment. TPT-related hearing dysfunction mainly affected the high and middle frequencies (115). Clinical and audiological evaluation both at baseline and during treatment is a reasonable monitoring strategy, although the management of TPT-related hearing dysfunction is still unclear.

TPT represents the first pharmacological treatment of GO which offers all-round efficacy (in terms of disease activity, severity and QoL) and it is effective regardless of the disease duration across a wide spectrum of GO, from inactive to active moderate-severe diseases and possibly sight-threatening dysthyroid optic neuropathy as well (117). TPT has become the first drug approved by the US Food and Drug Administration (FDA) for the treatment of adult GO since January 2020. ATA/ETA also recommends TPT as first-line treatment in active or progressive disease with significant proptosis or diplopia (50). Its safety profile is overall favorable but potential limitations in clinical application include issue of hearing dysfunction, relatively restricted geographical availability, and high cost (99).

### Linsitinib

#### Background and scientific basis

Linsitinib is an oral small molecule kinase inhibitor of IGF-1R and insulin receptor. In a mouse model, linsitinib effectively prevented development and progression of GO in terms of macrophage and T cell infiltration, inflammation and adipogenesis (118). By suppressing PI3K/Akt and extracellular signal-regulated kinase (ERK) pathways, linsitinib inhibited insulin-like growth factor 1 (IGF-1) induced cellular proliferation and HA secretion of GO-OF (119). Linsitinib induced apoptosis and inhibited proliferation of both IGF-1R and TSH-R expressing target cells in another *in-vitro* study (120).

#### Summary of key clinical trials and clinical application

The efficacy and safety of linsitinib in 90 patients with active moderate-to-severe GO was evaluated in a phase 2b/3, multicenter, randomized, double-masked, placebo-controlled study with proptosis reduction as its primary endpoint. Patients were randomized to linsitinib (75mg/150mg twice daily) or placebo for 24 weeks. Its positive topline results have recently been announced (121). Linsitinib at 150mg twice daily achieved significant proptosis response rate of 52% at week 24 (p = 0.01). No drug related hearing dysfunction was reported, and 1 of 29 patients (3%) developed hyperglycemia requiring no intervention. The full trial results will be released in due course and the confirmatory phase 3 study will commence in 2025. The advantage of oral administration, together with promising clinical efficacy and favorable safety profile, makes linsitinib an attractive therapeutic option for patients with GO.

### Batoclimab

#### Background and scientific basis

Immunoglobulin G (IgG) is an essential component of our adaptive humoral immunity against infection. Compared with other immunoglobulins, IgG is characterized by high circulating level, long half-life, and ability to move across mucosal surface and placenta. These properties are conferred by interactions with neonatal fragment crystallizable receptor (FcRn). FcRn is expressed in a diverse variety of body tissues, and it is predominantly located in the intracellular compartment with the highest concentration inside acidic endosomes. IgGs first enter the cells by pinocytic uptake as they cannot bind to FcRn at neutral pH on the cell surface. The formation of an early acidic endosome facilitates binding of FcRn to the Fc portion of IgGs. FcRn-bound IgGs are protected from lysosomal degradation. The complexes are diverted into recycling endosomes and exocytosed within exosomes, where IgGs are released from FcRn at neutral pH. When the interaction between FcRn and IgGs becomes saturated, the unbound IgGs will be subjected to lysosomal degradation. As a result, FcRn-mediated recycling prolongs the half-life of IgGs to around 20-23 days and help maintain their high circulating levels (122, 123). FcRn blockade becomes an attractive strategy to reduce circulating levels of pathogenic autoantibodies and FcRn inhibitors have shown positive effects in various autoimmune hematological and neurological conditions. Of note, selected FcRn inhibitors have already received regulatory approval for the treatment of immune thrombocytopenia purpura and generalized myasthenia gravis (123, 124).

#### Summary of key clinical trials and clinical application

Batoclimab (BTC) is a selective, fully human monoclonal antibody with high affinity for the IgG binding site on FcRn. In a proof-of-concept (POC), phase 2a, open-label single-arm trial (125), 7 patients with active moderate-to-severe GO received subcutaneous BTC 680mg weekly for 2 weeks, followed by 340mg weekly for 4 weeks. The primary endpoint was the change in TSH-R-Ab levels. There were significant reversible reductions in total TSH-R-Ab and TSAb levels during the treatment, which reached nadir by week 3 post-baseline and increased toward baseline level after BTC withdrawal.

The subsequent multicenter, randomized, double blind, placebo-controlled, phase 2b trial evaluated the efficacy and safety of 3 BTC dosing regimens (255/340/680mg weekly for 12 weeks) in patients with active moderate-to-severe GO (125). The primary endpoint was the proptosis responder rate (≥2mm reduction) at 12 weeks post-baseline. Initially the trial planned to include 77 patients, however it was paused and subsequently terminated due to hypercholesterolemia as an unexpected drug-related adverse event. An interim analysis was performed when 65 patients were randomized and included in the safety and intention-to-treat populations. At the time of trial pause, 45 patients (69%) completed the 12-week treatment and 2 withdrew. 18 (28%) patients discontinued study medication because of trial pause. 44 of 65 (68%) patients completed the 7-week follow-up.

Consistent with the findings from the POC trial, BTC resulted in an early, remarkable and dose-dependent reduction (up to >60%) in both total TSH-R-Ab and TSAb levels with a nadir at 12 weeks post-baseline when compared to placebo. The 3 TSH-R-Ab binding and bioassays (luciferase, cAMP) showed high correlation, and all were strongly and significantly correlated with CAS and proptosis (126). Significantly greater proportion of patients in the 2 higher dose groups (340/680mg) achieved proptosis response at multiple timepoints, however the difference does not reach statistical significance at 12 weeks post-baseline (i.e. pre-defined primary endpoint; ~30% vs 5% in placebo). The discrepancy was most likely explained by the incomplete clinical assessment due to trial discontinuation and COVID restrictions. There were also significantly more CAS responders (score 0 or 1) in BTC group at week 7 (255mg) and week 11 (680mg). No change in Gorman score for diplopia was noted. The low baseline prevalence of diplopia (40%) among participants and the imbalance of diplopia between BTC and placebo groups (55% vs 72%) may limit the detection of beneficial treatment effect. The EUGOGO GO-QoL appearance subscale significantly improved at 19 weeks post-baseline in BTC 680mg group. Only the two higher dose groups (340/680mg) demonstrated clinically relevant improvement (≥ 6 points) of both GO-QoL total score and appearance subscale at both 12 and 19 weeks’ post-baseline. Paired CT scans were available in 11 patients, and significant dose-dependent reduction in orbital muscle volume was observed at 12 weeks post-baseline in the 2 higher dose groups (340/680mg) compared with placebo. BTC was associated with significant reduction in thyroid hormones, although its effect on GH could not be independently assessed due to the concomitant use of ATD.

Most treatment-related adverse events were mild or moderate. There was no death or permanent discontinuation of study medication due to adverse events. There were no significant changes in blood cell counts, complement factors and liver enzymes. Peripheral edema was reported in five patients in BTC 680mg group, which resolved spontaneously in four cases despite continuation of study medication. Two special adverse events deserved attention. Reversible dose-dependent decline in serum albumin level (nadir at 7 weeks) and up to 59% increase in low-density lipoprotein cholesterol (LDL-C; peak at 7 weeks) were evident in BTC groups, and both abnormalities resolved within 8 weeks after treatment discontinuation. The reduction of serum albumin negatively correlated with the rise of total cholesterol and LDL-C (127). FcRn-mediated recycling and transcytosis rescue both albumin and IgG antibodies from intracellular lysosomal degradation, although their binding sites on FcRn are distinct and do not overlap (128). Therefore, albumin catabolism is accelerated in the presence of FcRn inhibitors. Hypercholesterolemia is a typical feature of patients with significant hypoalbuminemia (e.g. nephrotic syndrome). The proposed mechanisms of this phenomenon include: hepatic overproduction of apolipoprotein B (129); reduced metabolism of acetyl-CoA and more cholesterol synthesis by 3-hydroxy-3-methyl-glutaryl-CoA reductase (HMG-CoA reductase); and redistribution of albumin-bound fatty acids onto lipoproteins (127). As FcRn inhibitor-related hypercholesterolemia is fully reversible upon treatment withdrawal, its impact on cardiovascular risk is expected to be clinically insignificant if the treatment duration is short. Overall BCT is safe and well tolerated. Based on the promising findings from the above pilot RCT, the upcoming phase 3 randomized placebo-controlled trials (NCT05524571 and NCT05517421) and the associated open-label extension study (NCT05517447) will further define the efficacy, safety, and optimal regimen of BTC in the management of active moderate-to-severe GO.

**Figure 2:**
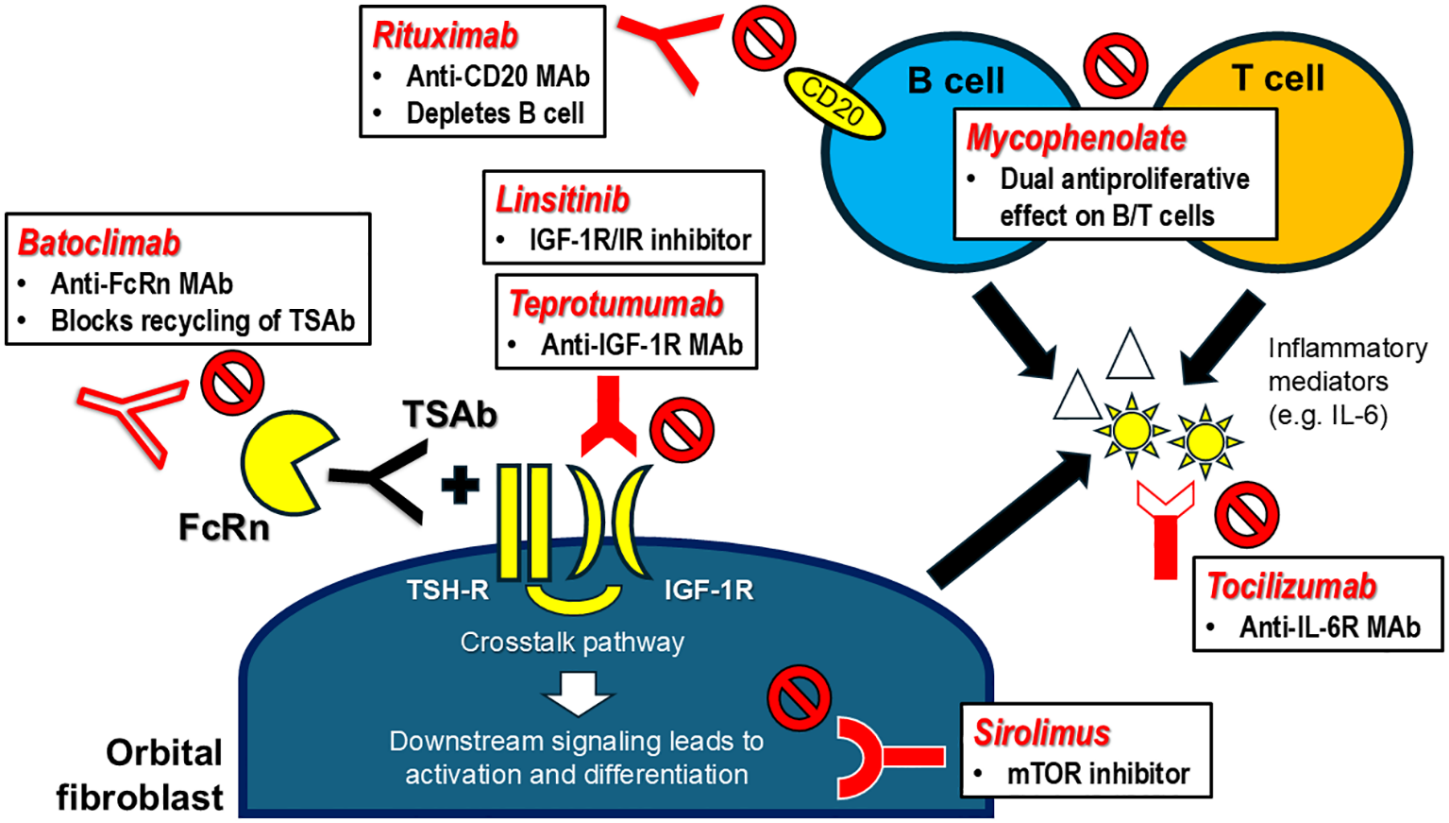
Targeted immunotherapies for Graves’ orbitopathy – mechanisms of action. FcRn, neonatal fragment crystallizable receptor; IGF-1R, insulin-like growth factor-1 receptor; IL-6, interleukin-6; IL-6R, interleukin-6 receptor; IR, insulin receptor; MAb, monoclonal antibody; mTOR, mammalian target of rapamycin; TSAb, thyrotropin receptor stimulating antibody; TSH-R, thyrotropin receptor.

The key clinical trials and mechanisms of action of targeted immunotherapies for GO are summarized in (**Table 2** and **Figure 2**), respectively.

**Table 2:** Targeted immunotherapies for Graves’ orbitopathy: summary of key clinical trials.

Therapeutics	Mechanism of action	Study Design/Patient population	Key findings	Comments/Important toxicities
Mycophenolate	Dual antiproliferative effect on B/T-cells via inhibiting IMPDH	◼ Multicenter RCT (53) ◼ 164 patients with active moderate-to-severe GO ◼ MPS 720mg daily + Weekly IVGC 4.5g vs Weekly IVGC 4.5g	Combination group demonstrated: ◼ 71% response rate at week 24 (vs 53%) ◼ 67% sustained response at week 36 (vs 43%)	◼ No difference in primary endpoints (response rate at week 12; relapse rates at week 24/36) between groups ◼ Combination group reported slightly more gastrointestinal intolerance but showed no increased risks of serious toxicities (e.g. serious infection, hepatotoxicity, cytopenia)
Rituximab(Italian)	Anti-CD20 MAb	◼ Single-center RCT (70) ◼ 32 euthyroid patients with active moderate-to-severe GO ◼ RTX (2g/500mg IV) vs weekly IVGC 7.5g	RTX group demonstrated: ◼ 100% CAS response (vs 69% in placebo) ◼ 0% recurrence (vs 33%) ◼ Better ocular dysmotility and GO-QoL visual functioning subscale score ◼ Less requirement for rehabilitative surgery	◼ Another RCT conducted in the US showed that RTX was not superior to placebo (71) ◼ Several patient characteristics in the Italian trial may explain its positive results (younger age, lower TSH-R-Ab and shorter disease duration) ◼ Risk of precipitating DON due to cytokine release syndrome
Tocilizumab	Anti-IL-6R MAb	◼ Multi-center RCT (89) ◼ 32 euthyroid patients with glucocorticoid resistant active moderate-to-severe GO ◼ TCZ (8mg/kg IV once every 4 weeks for 4 doses) vs placebo	Significantly more patients in TCZ group showed: ◼ CAS response: ≥2 CAS reduction (87-93% vs 59% in placebo at week 16/40); CAS <3 (80-87% vs 35-47% at week 16/40) ◼ GO-QoL improvement (47% vs 35%)No difference in proptosis/diplopia between the 2 groups	◼ A systematic review and meta-analysis of 12 studies (1 RCT, 11 cohort studies) showed that TCZ resulted in significant improvement in CAS, proptosis, diplopia, and TSH-R-Ab levels in patients with glucocorticoid resistant active moderate-to-severe GO ◼ Common side effects: mild neutropenia, hypercholesterolemia, transient rise in liver enzymes
Sirolimus	mTOR inhibitor	◼ Observational study (97) ◼ 30 patients with glucocorticoid resistant active moderate-to-severe GO ◼ Sirolimus (2mg as loading then 0.5mg daily for 12 weeks) vs weekly IVGC 4.5g	At week 24, significantly more patients in sirolimus group showed: ◼ Overall response (86.6% vs 26.6%) ◼ Proptosis response (80% vs 13.3%) ◼ CAS response (86.6% vs 33.3%)	◼ All treatment outcomes (except for CAS response) did not differ between the 2 groups at week 48
Teprotumumab	Anti-IGF1-R blocking MAb	◼ 3 multi-center RCTs* ◼ 221 euthyroid patients with active moderate-to-severe GO in total ◼ TPT (once every 3 weeks for 8 IV infusions; 1^st^ dose 10mg/kg, remaining 7 doses 20mg/kg) vs placebo	At week 24, significantly more patients in TPT group showed ^: ◼ Overall responders (73-89% vs 11-14%) ◼ CAS 0/1 (59-62% vs 22%) ◼ Proptosis response (77-89% vs 11-15%) ◼ Diplopia response (68% vs 27%) ◼ Diplopia resolution (50-53% vs 20-25%) ◼ Better GO-QoL scales	◼ All-round efficacy regardless of disease duration across a wide spectrum of disease phenotypes ◼ Common side effects: • muscle spasm • hyperglycemia (risk factors: older age, pre-existing diabetes, Asian/Hispanic) • hearing dysfunction (risk factors: older age, baseline hearing loss) ◼ Regular glycemic and audiological monitoring recommended during treatment
		◼ Open-label extension study (106) ◼ Patients in previous RCT who received placebo (n =37), did not respond (n = 5), or flared (n = 8) ◼ Same TPT regimen as previous RCTs	◼ 33/37 proptosis response ◼ 2/5 proptosis response ◼ 5/8 responded again	
		◼ Extended follow-up outcome analysis (107) ◼ 112 patients from 3 trials	◼ TPT displayed durable response ◼ At 1 year after the last dose of TPT, 91.2% (vs 86.6% at week 24), 89.5% (vs 91.1%), 72.9% (vs 70.2%), 67.9% (vs 86.6%), and 66.1% (vs 76.8%) of patients were responders for CAS, composite outcome, diplopia, proptosis, and overall response, respectively. ◼ Over 2 years following TPT therapy, 18% of patients underwent additional GO therapy	
		◼ RCT (109) ◼ 62 patients with proptosis, long GO duration (2-10 years) and low CAS ≤1 ◼ TPT vs placebo	◼ Proptosis response in TPT group 62% (vs 25% in placebo)	
Batoclimab	Anti-FcRn MAb	◼ Multicenter phase 2 RCT (125) ◼ 65 patients with active moderate-to-severe GO ◼ BCT (3 regimens: 255/340/680mg weekly sc for 12 weeks) vs placebo	◼ BCT resulted in early, marked, dose dependent, and reversible reduction (up to >60%) in both total TSH-R-Ab and TSAb ◼ Significantly more patients in BCT group (especially higher doses) showed:• Proptosis response at multiple timepoints • CAS response • GO-QoL improvement	◼ Common side effects include reversible, dose dependent reduction in both serum albumin level and LDL-C

BTC, batoclimab; CAS, clinical activity score; DON, dysthyroid optic neuropathy; FcRn, neonatal fragment crystallizable receptor; GO, Graves’ orbitopathy; GO-QoL, Graves’ orbitopathy quality of life questionnaire; IGF-1R, insulin-like growth factor-1 receptor; IL-6R, interleukin-6 receptor; IMPDH, inosine monophosphate dehydrogenase; IV, intravenous; IVGC, intravenous glucocorticoid; LDL-C, low-density lipoprotein cholesterol; MAb, monoclonal antibody; MPS, mycophenolate sodium; mTOR, mammalian target of rapamycin; RCT, randomized controlled trial; RTX, rituximab; TCZ, tocilizumab; TPT, teprotumumab; TSH-R-Ab, thyrotropin receptor antibody.

*refers to the following trials (103, 104, 110).

^pooled data from all 3 RCTs (103–105, 110):.

## Conclusions

Targeted immunotherapies have started to revolutionize our principles and approaches in the management of thyroid autoimmunity. Novel therapeutics which target the underlying immune dysregulation and TSH-R-Ab/TSH-R interactions may prove to be superior to inhibition of thyroid hormone synthesis by ATD in the setting of GH, when their clinical trials enter later phases of development. As a non-specific systemic immunosuppressant, glucocorticoid has been established as a standard treatment of GO for decades. Over the past 10 years, new therapeutic strategies have been developed to enhance treatment efficacy, including combination therapy (IVGC with mycophenolate or statin) and the application of immunotherapies, which target various specific pathogenic mechanisms. Teprotumumab represents a breakthrough due to its all-round efficacy, which allows extension of clinical benefits across a wider spectrum of disease phenotypes. The heated race for new treatments for GO with multiple upcoming drug trials will continue to optimize our management GO in an effective and safe manner (130).

## Glossary

ATA: American Thyroid Association
ATD: Antithyroid drug
BTC: Batoclimab
CAS: Clinical activity score
CD40L: CD40 ligand (CD154)
DON: Dysthyroid optic neuropathy
ERK: Extracellular signal-regulated kinase
ETA: European Thyroid Association
EUGOGO: European Group on Graves’ Orbitopathy
FcRn: Neonatal fragment crystallizable receptor
FDA: Food and Drug Administration
FU: Follow-up
GD: Graves’ disease
GH: Graves’ hyperthyroidism
GO: Graves’ orbitopathy
GO-QoL: Graves’ orbitopathy quality of life questionnaire
HA: Hyaluronic acid
HMG-CoA: 3-hydroxy-3-methyl-glutaryl-CoA
IGF-1: Insulin-like growth factor 1
IGF-1R: Insulin-like growth factor-1 receptor
IgG: Immunoglobulin G
IL-6: Interleukin-6
IL-6R: Interleukin-6 receptor
IM: Intramuscular
IMPDH: Inosine monophosphate dehydrogenase
IR: Insulin receptor
IV: Intravenous
IVGC: Intravenous glucocorticoid
LDL-C: Low-density lipoprotein cholesterol
MAb: Monoclonal antibody
MHC Class II: Major histocompatibility class II molecule
MPA: Mycophenolic acid
MPS: Mycophenolate sodium
mTOR: Mammalian target of rapamycin
OF: Orbital fibroblast
PI3K: Phosphatidylinositol 3-kinase
POC: Proof-of-concept
RAI: Radioactive iodine
RCT: Randomized controlled trial
RTX: Rituximab
TCR: T cell receptor
TCZ: Tocilizumab
TPT: Teprotumumab
TSAb: Thyrotropin receptor stimulating antibody
TSH: Thyrotropin
TSH-R: Thyrotropin receptor
TSH-R-Ab: Thyrotropin receptor antibody
